# The PPAR-γ agonist pioglitazone modulates inflammation and induces neuroprotection in parkinsonian monkeys

**DOI:** 10.1186/1742-2094-8-91

**Published:** 2011-08-05

**Authors:** Christine R Swanson, Valerie Joers, Viktoriya Bondarenko, Kevin Brunner, Heather A Simmons, Toni E Ziegler, Joseph W Kemnitz, Jeffrey A Johnson, Marina E Emborg

**Affiliations:** 1 Wisconsin National Primate Research Center, University of Wisconsin, Madison, WI USA; 2 Neuroscience Training Program, University of Wisconsin, Madison, WI USA; 3 Department of Physiology, University of Wisconsin, Madison, WI USA; 4 School of Pharmacy, University of Wisconsin, Madison, WI USA; 5 Department of Medical Physics, University of Wisconsin, Madison WI, USA

## Abstract

**Background:**

Activation of the peroxisome proliferator-activated receptor gamma (PPAR-γ) has been proposed as a possible neuroprotective strategy to slow down the progression of early Parkinson's disease (PD). Here we report preclinical data on the use of the PPAR-γ agonist pioglitazone (Actos^®^; Takeda Pharmaceuticals Ltd.) in a paradigm resembling early PD in nonhuman primates.

**Methods:**

Rhesus monkeys that were trained to perform a battery of behavioral tests received a single intracarotid arterial injection of 20 ml of saline containing 3 mg of the dopaminergic neurotoxin 1-methyl-4-phenyl-1,2,3,6-tetrahydropyridine (MPTP). Twenty-four hours later the monkeys were assessed using a clinical rating scale, matched accordingly to disability, randomly assigned to one of three groups [placebo (n = 5), 2.5 (n = 6) or 5 (n = 5) mg/kg of pioglitazone] and their treatments started. Three months after daily oral dosing, the animals were necropsied.

**Results:**

We observed significant improvements in clinical rating score (*P *= 0.02) in the animals treated with 5 mg/kg compared to placebo. Behavioral recovery was associated with preservation of nigrostriatal dopaminergic markers, observed as higher tyrosine hydroxylase (TH) putaminal optical density (*P *= 0.011), higher stereological cell counts of TH-ir (*P *= 0.02) and vesicular monoamine transporter-2 (VMAT-2)-ir nigral neurons (*P *= 0.006). Stereological cell counts of Nissl stained nigral neurons confirmed neuroprotection (*P *= 0.017). Pioglitazone-treated monkeys also showed a dose-dependent modulation of CD68-ir inflammatory cells, that was significantly decreased for 5 mg/kg treated animals compared to placebo (*P *= 0.018). A separate experiment to assess CSF penetration of pioglitazone revealed that 5 mg/kg p.o. induced consistently higher levels than 2.5 mg/kg and 7.5 mg/kg. p.o.

**Conclusions:**

Our results indicate that oral administration of pioglitazone is neuroprotective when administered early after inducing a parkinsonian syndrome in rhesus monkeys and supports the concept that PPAR-γ is a viable target against neurodegeneration.

## Background

Peroxisome proliferator-activated receptors (PPARs) are ligand-dependent transcription factors. Activation of the PPAR-γ subtype is known to increase insulin sensitization, modulate glucose and lipid metabolism. Pioglitazone (Actos^®^; Takeda Pharmaceuticals Ltd.) is a thiazoledinedione (TZD) and a highly selective PPAR-γ agonist. It is currently approved as an oral monotherapy and adjunctive therapy for patients with type 2 diabetes mellitus (T2DM; [1,2].

A growing body of evidence points towards chronic neuroinflammation having a key role in Parkinson's Disease (PD) pathogenesis [3-6]. This suggests that anti-inflammatory strategies may be beneficial to prevent PD's typical progressive loss of dopaminergic nigral neurons [7]. PPAR-γ activators reduce inflammation by inhibiting expression of proinflammatory cytokines and metalloproteases [8,9]. In a model of neuroinflammation by intrastriatal injection of lipopolysaccharides (LPS), pioglitazone decreased glial activation, improved mitochondrial function and attenuated oxidative stress, preserving nigral dopaminergic cell count and partially restoring striatal dopamine [10,11]. In mouse models of PD induced by 1-methyl-4-phenyl-1,2,3,6-tetrahydropyridine (MPTP) intoxication, oral administration of pioglitazone reduced glial activation and attenuated loss of substantia nigra (SN) pars compacta dopaminergic (DA) neurons, promoted lκBα induction and blocked NFκB and iNOS activation [12,13] In nonhuman primates (NHP) the most used models of PD are induced by MPTP administration [14] which, similar to mice, it induces loss of dopaminergic nigral cells and their striatal terminals as well as inflammation that persists many years after the original neurotoxin exposure [15-17]. Yet, the effects of PPAR-γ agonists in a NHP PD model have not been assessed and its investigation may define whether the clinical translation of this strategy is valid [18,19].

Here we report our evaluation of the disease modifying properties of pioglitazone in a paradigm resembling early PD in NHP. We hypothesized that the PPAR-γ agonist would modulate the inflammatory reaction induced by the neurotoxin MPTP and, in consequence, prevent nigral cell loss and associated PD syndrome. We chose to induce a hemiparkinsonian model by a single intracarotid artery administration of MPTP due to the stability and replicability of the model [20]. We started pioglitazone administration 24 hours after neurotoxin challenge to resemble the ongoing degeneration observed in PD patients [21,22]. Oral dosing was equivalent to the one used to treat diabetic conditions. Our results demonstrate that pioglitazone administration attenuated the inflammatory response, preserved dopaminergic nigrostriatal function and improved PD signs in this experimental paradigm.

## Methods

### Animals

Adult rhesus monkeys (*Macaca mulatta*, 5-7 years old) were obtained from the Wisconsin National Primate Center (WNPRC) and singly housed with a 12-hr light/dark cycle at the WNPRC facility. Purina monkey chow and water was available *ad libitum*. The animals' diet was supplemented with fruit during the testing sessions and daily enrichment. All efforts were made to minimize the number of animals used and ameliorate their suffering. This study was performed in strict accordance with the recommendations in the Guide for the Care and Use of Laboratory Animals of the National Institutes of Health. Two sets of experiments were carried out: a neuroprotective and a CSF penetration analysis. The protocols were approved by the Institutional and Animal Care Committee at the University of Wisconsin-Madison (permits #: G00492 and G00569, respectively).

### Behavioral Evaluations

All behavioral training and evaluations used positive reinforcement to entice monkeys' cooperation. Monkeys were evaluated weekly using a clinical rating (CR) scale as previously described [23,24]. The scale ranges from 0 to 32; a score of 0 corresponds to normal behavior and 32 to extreme severe parkinsonian symptoms. Fine motor skills (FMS) were tested using a movement assessment panel computerized system 3 days per week [24]. General activity was assessed pre-MPTP and once per month after MPTP using image digitization followed by computerized post-processing of animal movement (Viewpoint, Inc; [24]).

### Induction of PD syndrome

After baseline data collection was completed, 20 rhesus monkeys (male, 5-7 yrs, 4-8 kg) received a unilateral intracarotid artery injection of 3 mg of MPTP-HCl (Sigma) in 20 ml of saline (rate: 1.33 ml/min) as previously described [23]. The procedure was performed in a state-of-the-art surgical suite under isofluorane anesthesia (1-2%). Throughout the procedure vital signs were monitored and recorded. Each animal was given cefazolin (25 mg/kg i.m.) and buprenex (0.01 mg/kg i.m.) upon waking up response and 24 hours post surgery.

Twenty-four hours after MPTP administration, the 20 animals were behaviorally assessed with the CR scale and 16 monkeys were selected (CR score ≥ 9 points), matched according to PD signs, randomly assigned to one of three groups (see below) and their treatments started.

### Pioglitazone dosing

To assess for pioglitazone side effects in non-diabetic animals, one month before MPTP surgery a glucose tolerance test, general serum panel, glycosylated hemoglobin, and insulin levels were performed. Afterwards the animals received pioglitazone (5 mg/kg p.o.) once a day for 7 days and the tests repeated.

After 1 month of washout the animals were intoxicated with MPTP and 24 hours later animals were selected and treatments started. Once a day until the end of the study, the animals received oral dosing of vehicle, (n = 5), 2.5 (n = 6) or 5 mg/kg (n = 5) of pioglitazone [1]. During treatment animals were weighed weekly and serum samples were taken monthly. Before necropsy, a glucose tolerance test was performed. The animals were trained for blood sampling using positive reinforcement.

### Necropsy and tissue preparation

Three months post-MPTP the animals were anesthetized with sodium pentobarbital (25 mg/kg iv) and transcardially perfused with heparinized saline, followed by 4% paraformaldehyde (PFA; [23,24]). Brains were post-fixated in 4% PFA for 12-24 hours and cryoprotected by immersion in a graded (10-40%) sucrose/0.1 M phosphate buffered saline (PBS, pH 7.2) solution. The tissue was cut frozen (40 μm sections) on a sliding knife microtome. All sections were stored in a cryoprotectant solution before processing.

All other major organs were examined and sampled for histology at the time of necropsy. Tissues were fixed in 10% neutral buffered formalin, and routinely processed for hematoxylin and eosin staining.

### Immunohistochemistry

Brain coronal sections were stained with Nissl or by immunohistochemical methods according to our previously published protocols [23,24]. Antibodies used include: tyrosine hydroxylase (TH; 1:20,000; Immunostar, Hudson, WI), vesicular monoamine transporter 2 (VMAT2; 1:1000; Phoenix Pharmaceuticals, Belmont, CA), glial fibrillary acidic protein (GFAP; 1:2,000; DakoCytomation, Glostrup, Denmark), heme-oxygenase-1 (HO-1; 1:1,000; Assay Designs "Stressgen", Ann Arbor, MI), nitrotyrosine (1:300; Millipore, Billerica, MA), and CD68 (1:3,000; DakoCytomation, Glostrup, Denmark).

### Neuroanatomical Evaluation

The optical density (OD) of TH and VMAT2 immunoreactive (ir) striatal fibers was quantified within ventral, medial and dorsal sections of both the caudate and putamen using NIH ImageJ software. Images of nine coronal sections per monkey approximately 2 mm apart were captured using an Epson 1640XL-GA high-resolution digital scanner. ImageJ was calibrated using a step tablet, grey scale values were converted to OD units using the Rodbard function, and the mean OD for each area of interest was recorded.

The total number of TH-ir, VMAT2-ir, and Nissl neurons in the right and left substantia nigra (SN) was calculated using unbiased stereological cell-counting methods described previously [23,25-27]. The optical dissector system consisted of a computer assisted image analysis system including a Zeiss Axioplan 2 imaging photomicroscope (Carl Zeiss, Inc) hard-coupled to a MAC5000 high precision computer-controlled x-y-z motorized stage, and a MicroFire CX9000 camera (Optronics, Goleta, CA). Neuronal counts were performed using Stereo Investigator Version 7.5 (MicroBrightField, Williston, VT). The SN was outlined under a low magnification (2.5×). The total number of TH-ir and VMAT2-ir neurons within the counting frame was counted using a 100× oil immersion objective with a 1.4 numerical aperture. Six equally spaced sections from each subject containing the SN were used for analysis.

The amount of CD68, GFAP, HO-1, and nitrotyrosine immunoreactivity (ir) was quantified within the SN using NIH ImageJ software. Images from five coronal sections per monkey approximately 2 mm apart were captured using a Nikon E800 microscope equipped with a SPOT camera. CD68-ir was calculated using the particle count function of the ImageJ program, which refers to the quantification of objects of a certain size within a region of interest in a thresholded image. The object size was set between five and 75 square pixels. The particle number and their total area within each region of interest was then analyzed and recorded. For GFAP, nitrotyrosine and HO-1-ir, ImageJ was calibrated using a step tablet, grey scale values were converted to OD units using the Rodbard function, and the area in pixels above a threshold of 0.30 OD units was recorded.

### Analysis of plasma and CSF levels of pioglitazone

To determine CSF penetration of pioglitazone, five rhesus monkeys (female, 6-7 yrs, 5-6 kg) received daily oral administration of placebo, 2.5, 5 or 7.5 mg/kg of pioglitazone. Each dosing was given for a period of 8 days. Plasma and immediately thereafter, CSF samples were obtained at baseline and approximately 5 hours after the last dose of pioglitazone. CSF and blood collections were performed under ketamine (7 mg/kg i.m.) and medetomidine (0.05 mg/kg i.m.) anesthesia. Animal vital signs were monitored until they completely recovered. Samples were stored at -80°C until analysis.

Drug levels were evaluated by HPLC methods as described by Sripalakit et al. [28] with modifications. Plasma and CSF samples were stored at -80°C until analysis. To purify the samples, they were thawed and processed through solid phase extraction (SPE) based on a previously described method with modifications [28]. One ml of sample was processed with 1 ml KH_2_PO_4 _(0.1 M) and 400 μl rosiglitazone as the internal standard (Rosiglitazone-maleate 2 mg tablets, Avandia^®^, 10 μg/ml stock solution in acetonitrile (ACN)/buffer). The SPE columns (Strata C18-T, 100 mg/1 ml) were preactivated with 1 ml each of 100% ACN and then 1 ml KH_2_PO_4 _(0.1 M). Samples were added and then washed with 1 ml methanol-Kh_2_PO_4 _(30:70) and 1 ml K_2_HPO_4_. Columns were allowed to dry for 5 minutes and then were eluted by adding 500 μl ACN:H_2_O (40:60) and 500 μl ACN:H_2_O (50:50). For plasma samples, the elution was centrifuged at 3,000 for 5 minutes and transferred into HPLC vials. For CSF samples, the elution was dried under air at 60°C to concentrate the sample, resuspended in 250 μl of 50% CAN, and transferred into HPLC vials.

Pioglitazone standards were prepared as reported [28] with concentrations of 2,400, 1,200, 600, 300, 150, 75, 37.5, 18.75, 9.38, 4.69 ng diluted in 1 ml of plasma or CSF, 1.0 ml KH_2_PO_4 _with 400 μl internal standard. Standards were linear in plasma (*r^2 ^*= 0.996) and in CSF (*r^2 ^*= 0.997).

HPLC analysis used a Beckman HPLC system consisting of an automated sampler with a 100 μl loop, dual pumps, and a diode array analyzer for UV detection. Measurement was made at 269 nm wavelength. Chromatographic conditions and column were based on previously described methods and samples were injected as 100 μl. Retention times for rosiglitazone and pioglitazone were 4.3 and 10.1, respectively. Assay precision was 7.25% at 300 nag and 11.9% at 75 nag of pioglitazone (n = 9). Accuracy was 103.57% ± 2.53 (mean ± SEM).

### Data Analysis

Sample size was defined by *a priori *power analysis based on MPTP-treatment differences in motor function to achieve an alpha = 0.05 and beta < 0.2 (power > 80%). All data was collected and analyzed by investigators blind to the treatment groups. A *P *value of < 0.05 was considered statistically significant. All statistics were performed using SPSS version 17.0 software (SPSS, Chicago, IL).

## Results

### Pioglitazone improves functional measures of parkinsonism

**Figure 1:**
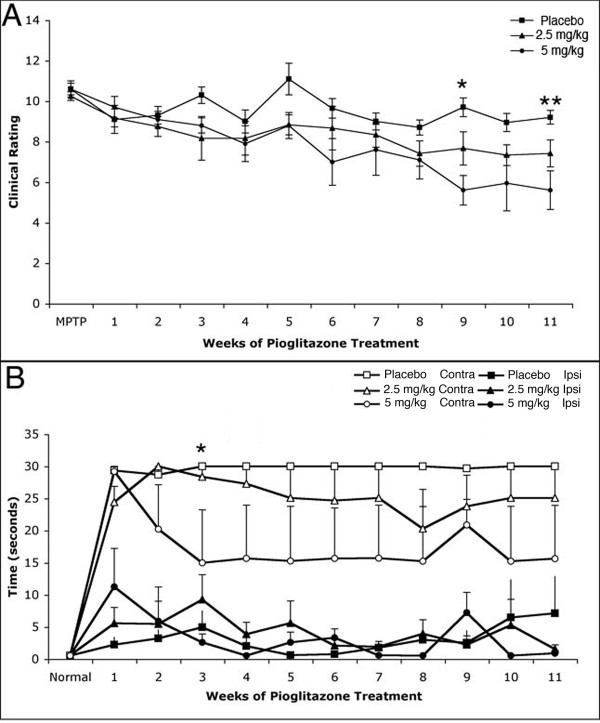
**Clinical Rating Score and Fine Motor Skills task**. (A) Clinical rating showed a progressive improvement of the hemiparkinsonian features, which reached statistical significance at 9 and 11 weeks (Kruskal-Wallis test; *P = 0.014, **P = 0.02, respectively). (B) During the fine motor skills task, the placebo-treated monkeys failed to complete the test with the left hand (hand contralateral to intracarotid MPTP administration) while some pioglitazone- treated monkeys improved their performance. Due to individual variability, a statistical significant difference was found only in the third week post-treatment in the 5.0 mg/kg treatment group compared to placebo (*Repeated measures ANOVA; *P *= 0.023).

Before MPTP treatment all monkeys showed no neurological impairments, scoring zero in the CR (Figure 1-A). Twenty-four hrs after intoxication, the animals presented a typical hemiparkinsonian syndrome, consisting of tremors in the side contralateral to MPTP infusion, slowness and decreased amount of movement, as well as balance and gait impairments (score ≥ 9 points). Over time the pioglitazone-treated monkeys showed a progressive improvement, in particular, bradykinesia, gross motor skills and gait that reached statistical significance for the 5 mg/kg treated monkeys compared to placebo at 9 (mean ± SEM, 5 mg/kg pioglitazone 5.60 ± 0.73, placebo 9.70 ± 0.46) and 11 weeks (5 mg/kg pioglitazone 5.60 ± 0.95; placebo 9.20 ± 0.34) (Kruskal-Wallis test; *X ^2 ^*= 8.500, df = 2, *P *= 0.014 and *X ^2 ^*= 7.833, df = 2, *P *= 0.02, respectively).

Pioglitazone's effect on FMS testing was more variable. Prior to MPTP administration, all subjects were able to complete the FMS task consistently and quickly with both hands (Figure 1-B). After MPTP administration, most monkeys had difficulty in completing the FMS task under 30 seconds using the hand contralateral to MPTP dosing (left hand), while exhibiting no deficits in task performance using the hand ipsilateral to MPTP administration (right hand). Two of the pioglitazone-treated monkeys in the 5 mg/kg group showed improvement in FMS. Due to individual variability, a statistically significant difference was found only in the third week post-treatment using the hand contralateral to MPTP dosing (repeated measures ANOVA; F [2,12] = 3.797, *P *= 0.04). *Post hoc *analysis further confirmed the observation that monkeys treated with 5 mg/kg of pioglitazone had significantly shorter latency times compared to placebo (Fisher's LSD *P *= 0.023). Performance with the hand ipsilateral to MPTP administration showed no significant differences between treatment groups over time (repeated measures ANOVA; F [2,12] = 0.771, *P *= 0.701).

The overall amount of activity of all monkeys was measured using a digitized monitoring system. No evidence of pioglitazone-induced hyperkinesia was observed (Table 1). The placebo monkeys showed an increase in activity in the first month after MPTP, probably due to a disbalance in brain DA that induced periods of spontaneous circling to the side of the brain lesion (observed as bursts of activity) but due to individual differences did not reach statistical significance. Repeated measures ANOVA failed to find in the activity data a significant effect of time (F [1,12] = 1.885, *P *= 0.195), treatment (F [2,12] = 1.767, *P *= 0.213) or an effect of time × treatment (F [2,12] = 1.462, *P *= 0.270).

**Table 1:** Duration of burst activity

Treatment Group	1 month Post-MPTP	2 months Post-MPTP	3 months Post-MPTP
Placebo	0.4 ± 0.322	29.5 ± 28.323	2.2 ± 0.776

2.5 mg/kg pioglitazone	0.4 ± 0.196	0.8 ± 0.385	0.6 ± 0.22

5 mg/kg pioglitazone	0.6 ± 0.21	0.9 ± 0.437	0.5 ± 0.409

Two months following MPTP administration, placebo-treated monkeys showed an increase in the duration of burst activity (expressed as the mean ± SEM of the ratio baseline/post period) compared to baseline. Due to individual differences repeated measures ANOVA; failed to find a significant effect of time (*P *= 0.195), treatment (*P *= 0.213) or effect of time × treatment (*P *= 0.270).

### Pioglitazone administration was well tolerated

Throughout the study, clinical and laboratory parameters were within range of normal adult male rhesus monkeys, including glucose levels. Following MPTP administration, all subjects presented a predictable loss of weight (approximately 0.5 kg). No significant differences in weight were observed between treatment groups (ANOVA; F [2,13] = 0.195, *P *= 0.825). Necropsy with histology of major organs of all monkeys did not show any remarkable finding.

### DA striatal fibers and nigral neurons were protected by pioglitazone

**Figure 2:**
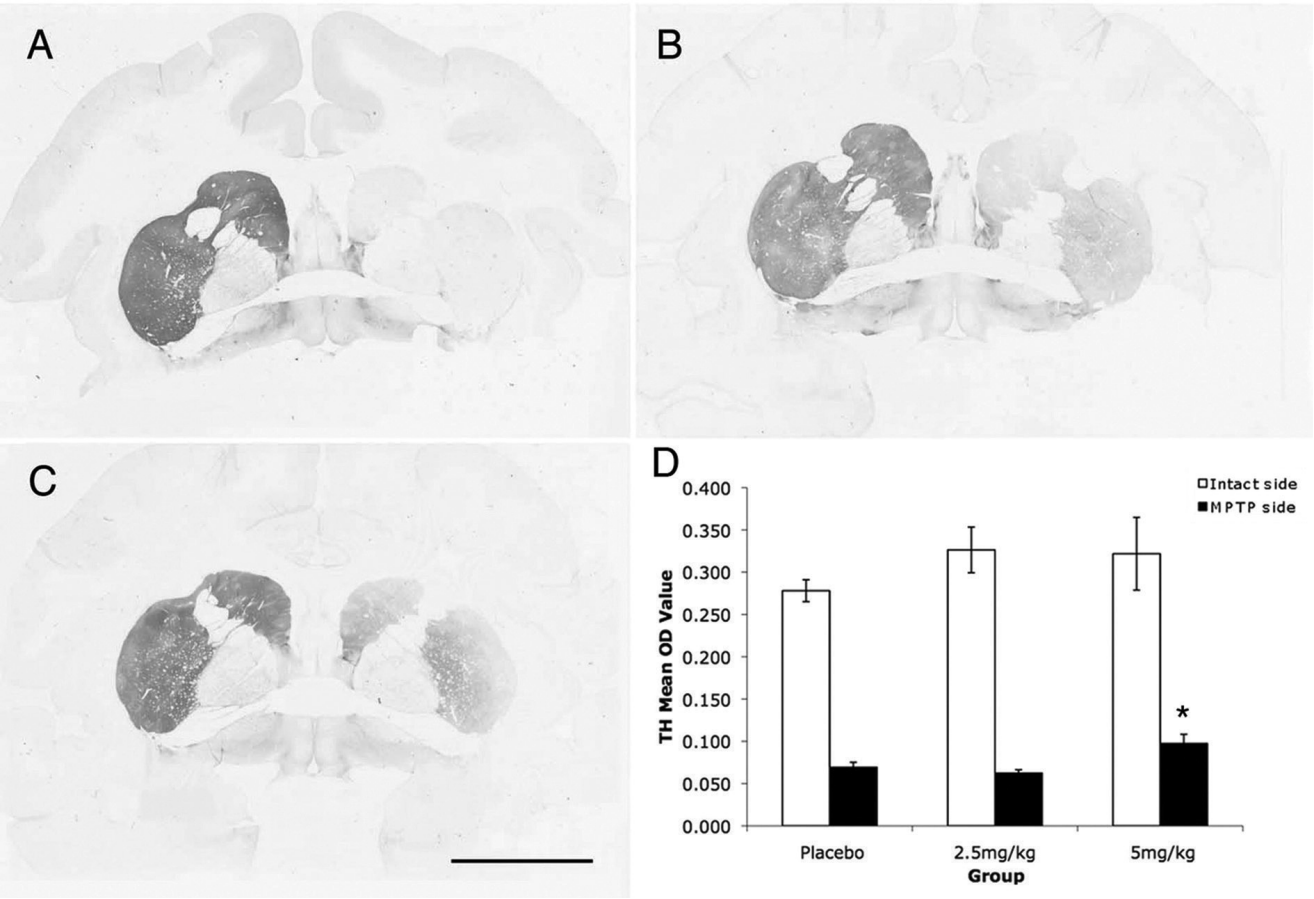
**Preservation of TH-ir fibers in the striatum**. Microphotographs of TH immunostained coronal brain sections at the level of the anterior commisure of (A) placebo (B) 2.5 mg/kg and (C) 5 mg/kg pioglitazone-treated hemiparkinsonian monkeys. Scale bar = 10 mm. Note the preservation of TH-ir fibers in the caudate and putamen ipsilateral to MPTP insult in pioglitazone-treated animals. (D) Optical density (OD) of TH immunostaining in the intact and MPTP treated putamen (*ANOVA; *P *= 0.001).

To assess DA striatal terminal fiber condition after MPTP and pioglitazone treatments analysis of TH and VMAT2 immunostaining were performed. OD quantification of TH-ir striatal fibers showed a significant loss on the side ipsilateral to MPTP administration compared to the contralateral side (Wilcoxon Signed Rank test; z = -3.408, *P *= 0.001, two tailed). There was a significant main effect of treatment between groups (ANOVA; F [2,143] = 11.76, *P = *0.012). Comparison between groups revealed that the ipsilateral putamen of the animals treated with 5 mg/kg of pioglitazone had a significant preservation of TH-ir fibers compared to placebo (ANOVA; Fisher's LSD *post hoc P = *0.001; Figure 2). OD quantification of VMAT2-ir striatal fibers also showed significant loss on the side ipsilateral to MPTP compared to the contralateral side (Wilcoxon Signed Rank test; z = -3.408, *P = *0.001). Although a slight preservation in the ipsilateral putamen for animals treated with pioglitazone was found, it did not reach statistical significance (ANOVA; F [2,12] = 0.555, *P = *0.25).

**Figure 3:**
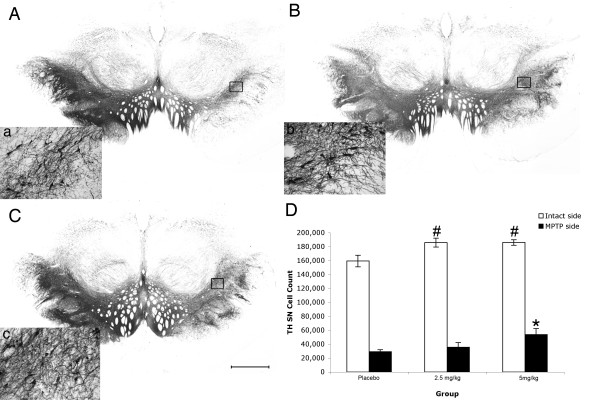
**Preservation of TH-ir cells in the substantia nigra**. Microphotographs of TH immunostained coronal brain sections at the level of the substantia nigra of placebo (A, a), 2.5 mg/kg (B, b), and 5 mg/kg (C, c) pioglitazone-treated hemiparkinsonian monkeys. Rectangles in A, B and C indicate the location of high magnification images a, b, c. Scale bar: A, B, C = 1 mm; a, b, c = 325 μm. D: Stereological cell counts showed a significant preservation of TH-ir cells in the substantia nigra ipsilateral to MPTP insult in 5 mg/kg pioglitazone-treated group compared to placebo (*ANOVA; *P *= 0.02), and higher TH-ir cell count in the intact side of animals treated with 2.5 and 5 mg/kg of pioglitazone compared to placebo (#ANOVA; *P *= 0.014, *P *= 0.02).

DA SN cell survival was evaluated by analysis of TH and VMAT2 immunohistochemistry, as well as Nissl histochemistry. Qualitatively, all subjects had preservation of neurons in the side contralateral to MPTP administration as well as in the ventral tegmental area. In the ipsilateral SN the placebo-treated monkeys showed decreased numbers of neurons and the surviving neurons had a shrunken perikarya and diminished neuropil. The pioglitazone monkeys instead, displayed more nigral neurons with a large round perikarya and extensive multipolar neurites. Stereological cell counts showed a significant loss of TH-ir nigral neurons on the side ipsilateral to MPTP administration compared to the contralateral side (Wilcoxon Signed Rank test; z = -3.408, *P *= 0.001 two-tailed). There was a main effect of treatment between groups (ANOVA; F [2,13] = 3.493, *P *= 0.05). Monkeys treated with 5 mg/kg of pioglitazone had higher cell counts (53,7345 ± 8,715) in the ipsilateral SN compared to controls (29,175 ± 2,954) (ANOVA; Fisher's LSD *post hoc P *= 0.02; Figure 3). Interestingly, the contralateral SN of both pioglitazone-treated groups also had a significantly higher number of nigral TH-ir cells (2.5 mg/kg, 185,987 ± 6420; 5 mg/kg, 186,064 ± 3,991) compared to controls (159,44 ± 8234) (ANOVA; Fisher's LSD *post hoc P *= 0.014, *P *= 0.02, respectively).

**Figure 4:**
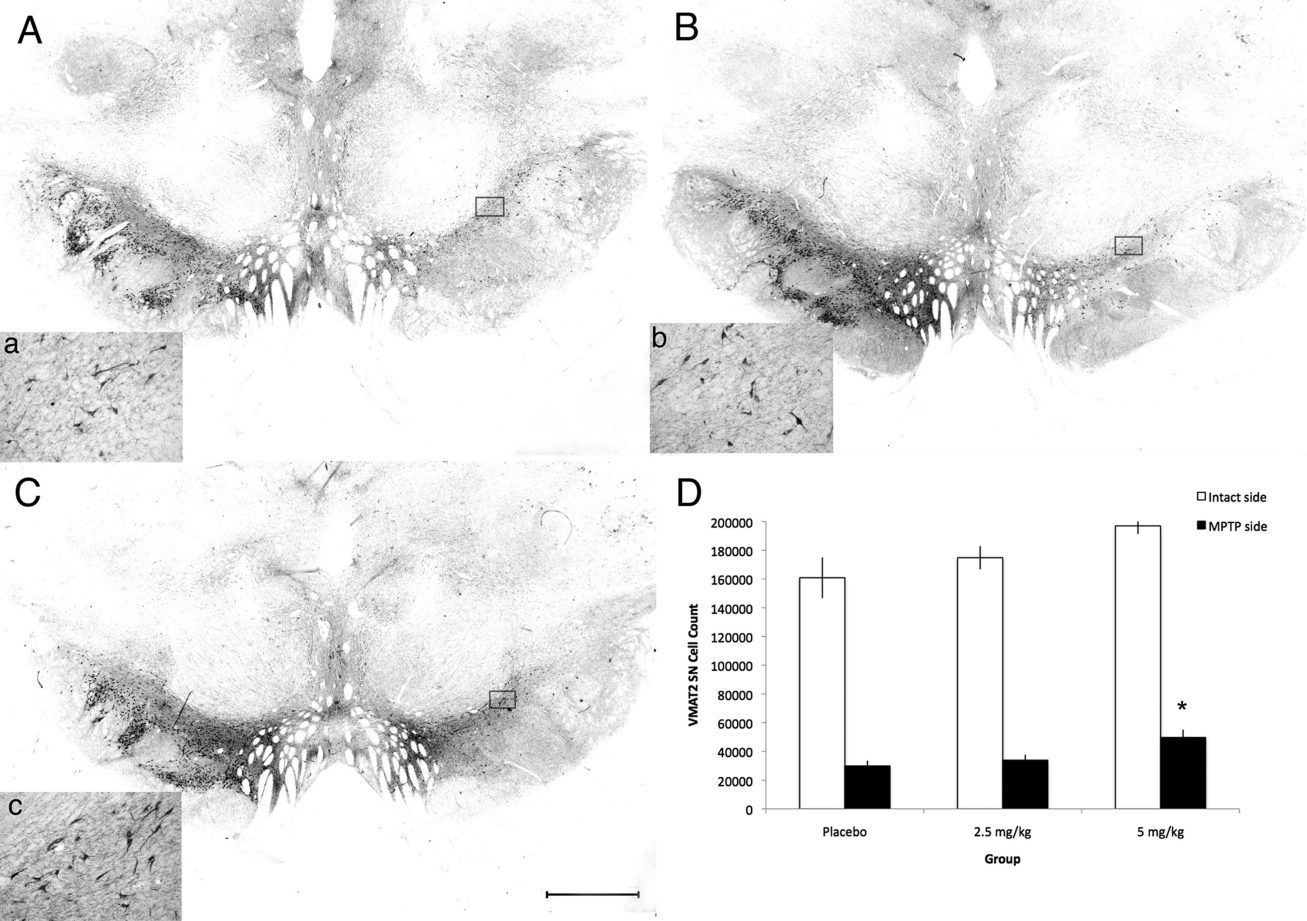
**Preservation of VMAT2-ir cells in the substantia nigra**. Microphotographs of VMAT2 immunostained coronal brain sections at the level of the substantia nigra of placebo (A, a), 2.5 mg/kg (B, b), and 5.0 mg/kg (C, c) pioglitazone-treated hemiparkinsonian monkeys. Rectangles in A, B and C indicate the location of high magnification images a, b, c. Scale bar: A, B, C = 1 mm; a, b, c = 325 μm. D: Stereological cell quantification showed a significant preservation of VMAT2-ir cells in the substantia nigra ipsilateral to MPTP insult in 5 mg/kg pioglitazone-treated group compared to placebo (*ANOVA; *P *= 0.04).

VMAT2-ir nigral neuron stereological quantification showed a significant loss on the side ipsilateral to MPTP administration compared to the contralateral side (Wilcoxon Signed Rank test; z = -3.408, *P *= 0.01 two-tailed). There was a main effect of treatment between groups (ANOVA; F [2,13] = 5.958, *P *= 0.016). A significant preservation of VMAT2-ir neurons in the SN ipsilateral to MPTP administration was found in animals treated with 5 mg/kg of pioglitazone (49,701 ± 5,391) compared to placebo (29,911 ± 3,631) (ANOVA; Fisher's LSD *post hoc P *= 0.04; Figure 4). No significant differences between treatment groups were observed in the contralateral SN.

**Figure 5:**
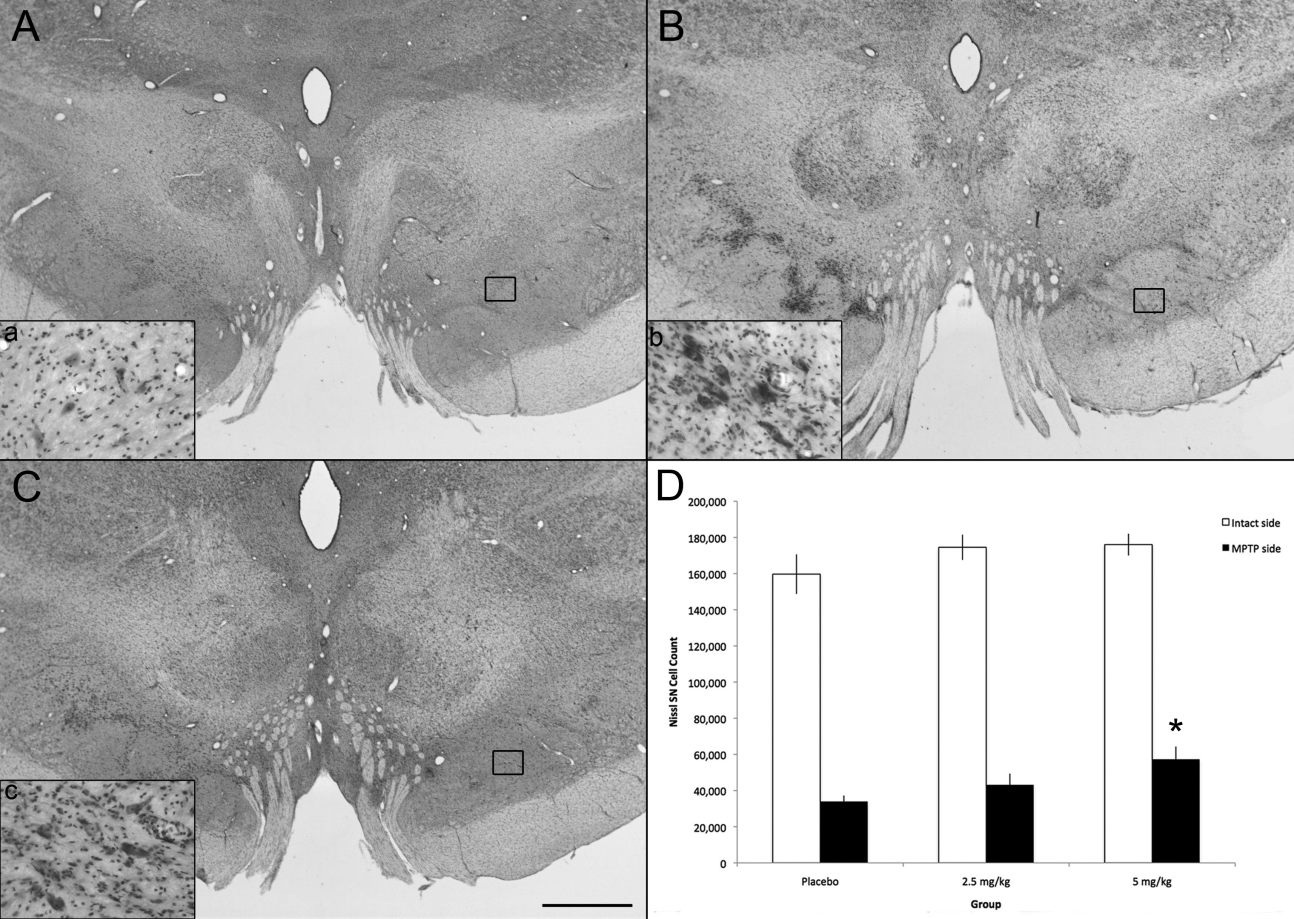
**Preservation of Nissl stained cells in the substantia nigra**. Microphotographs of Nissl stained coronal brain sections at the level of the substantia nigra of placebo (A, a), 2.5 mg/kg (B, b), and 5 mg/kg (C, c) pioglitazone-treated hemiparkinsonian monkeys. Rectangles in A, B and C indicate the location of high magnification images a, b, c. Scale bar: A, B, C = 2 mm; a, b, c = 50 μm. D: Stereological cell quantification showed a significant preservation of Nissl positive cells in the substantia nigra ipsilateral to MPTP insult in 5 mg/kg pioglitazone-treated group compared to placebo (*ANOVA; *P *= 0.007).

Nissl-stained nigral neuron stereological quantification confirmed a significant neuronal loss on the side ipsilateral to MPTP administration in comparison to the contralateral side (Wilcoxon Signed Rank test; z = -3.408, *P *< 0.001, two-tailed). There was a main effect of treatment between groups (ANOVA; F [2,12] = 5.414, *P *= 0.021). Monkeys treated with 5 mg/kg of pioglitazone had higher cell counts (57,297 ± 7,033) in the ipsilateral SN compared to controls (33,974 ± 3,275) (ANOVA; Fisher's LSD *post hoc P *= 0.017; Figure 5).

**Figure 6:**
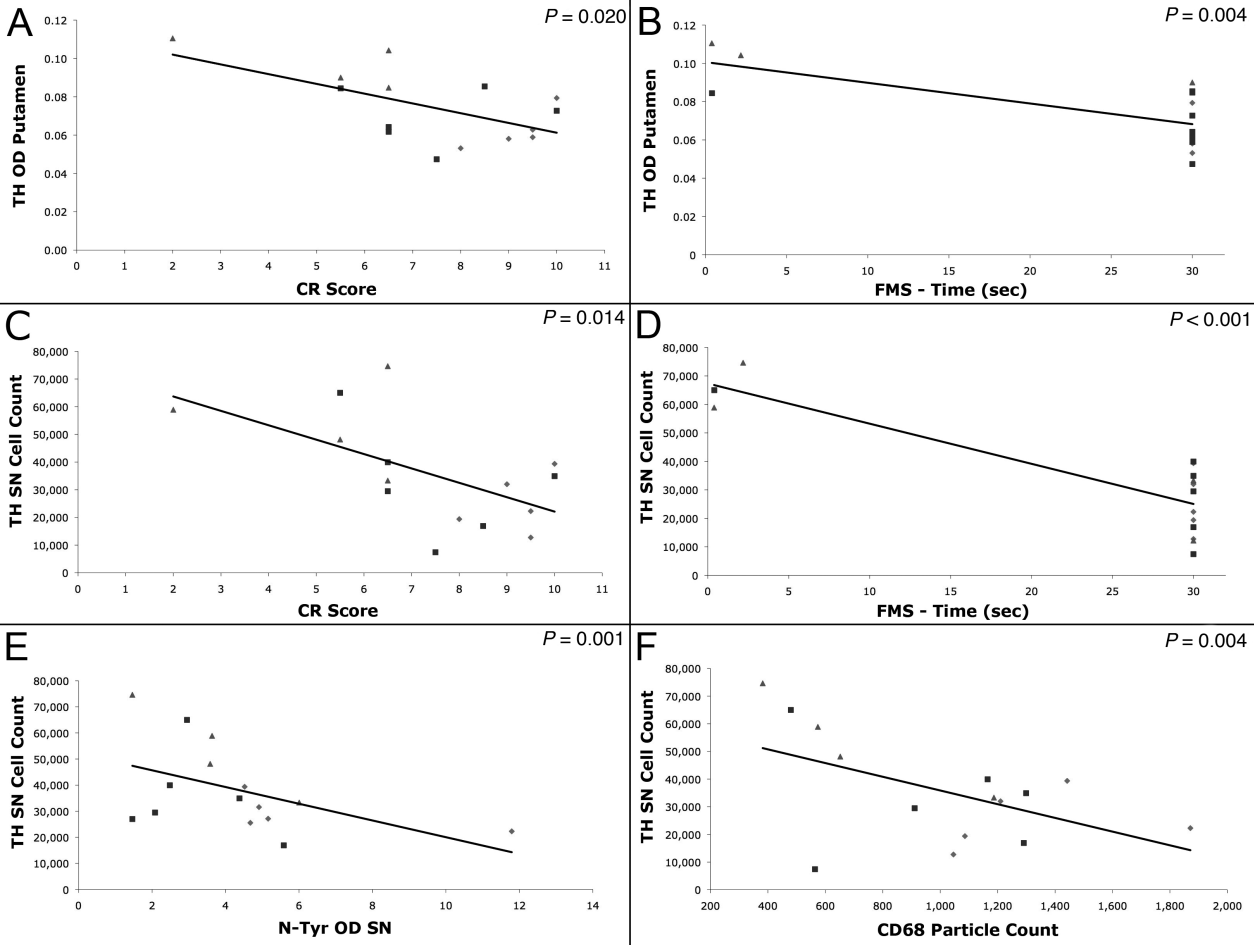
**Correlations between functional and anatomical markers**. Improvement in PD signs measured by clinical rating (CR) was associated with more TH-ir optical density (OD) in the putamen (Pearson's correlation; *P *= 0.0024) and higher number of TH-ir nigral cells (*P *= 0.002). Fine motor skill (FMS) performance also correlated with the amount of preservation of TH-ir putaminal fibers (*P *= 0.004) and nigral cells (*P *< 0.001). Optical density of nitrotyrosine (N-tyr) (P = 0.001) and CD68 (*P *= 0.014) immunostaining was negatively correlated with the number of TH-ir nigral cells. Diamond = placebo, square = 2.5 mg/kg pioglitazone, triangle = 5 mg/kg pioglitazone.

Measures of dopaminergic innervation correlated with behavioral performance (Figure 6). The animals that presented a lower score in the CR (indicating improvement in their PD signs) had more TH-ir OD in the putamen (Pearson's correlation; *r^2 ^*= 0.320, *P *= 0.002), as well as more TH (*r^2 ^*= 0.338, *P *= 0.024) and VMAT2 (*r^2 ^*= 0.689, *P *= 0.003) positive neurons in the SN. Similar to the CR, better performance in the FMS test (observed as less time needed to complete the task) inversely correlated with TH-ir fibers in the putamen (*r^2 ^*= 0.386, *P *= 0.004), as well as TH-ir (*r^2 ^*= 0.654, *P = *0.005) and VMAT2-ir (*r^2 ^*= 0.879, *P *= 0.004) neurons in the SN indicating that better performance in the task was associated with more DA markers in the nigrostriatal system. Additionally, the number of TH-ir nigral cells was positively correlated with the amount of TH-ir OD in the putamen (*r^2 ^*= 0.618, *P *= 0.001).

### Pioglitazone attenuated inflammation

**Figure 7:**
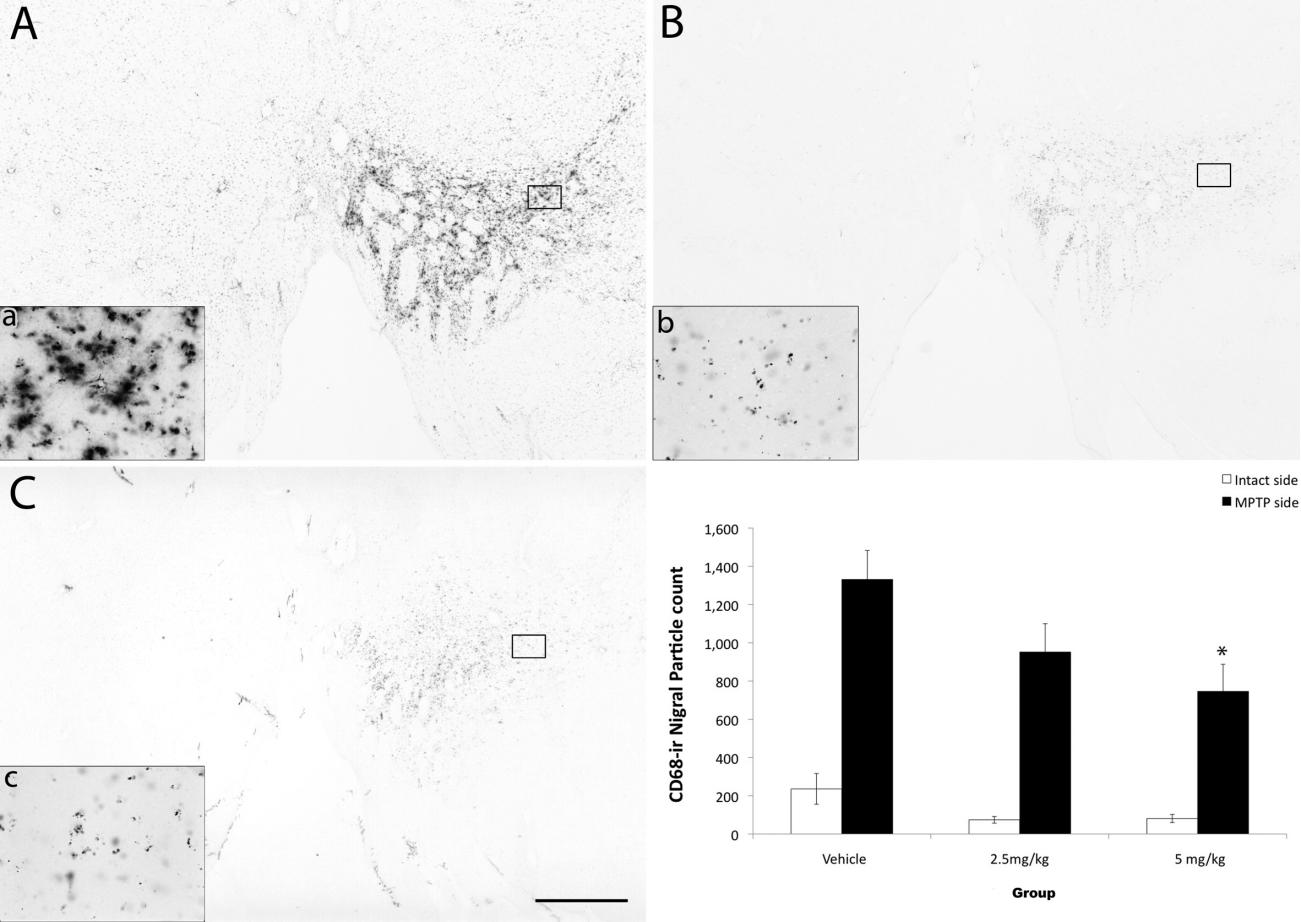
**Modulation of neuroinflammation in the substantia nigra**. Microphotographs of CD68 immunostained coronal brain sections, at the level of the substantia nigra of placebo- (A, a), 2.5 mg/kg (B, b), and 5 mg/kg (C, c) pioglitazone-treated hemiparkinsonian monkeys. Rectangles in A, B and C indicate the location of high magnification images a, b and c. Scale bar: A, B, C = 2 mm; a, b, c = 115 μm. Note the less intense microglial activation in the substantia nigra ipsilateral to MPTP insult in pioglitazone-treated groups. D: CD68-ir nigral quantification results. Analysis showed significantly less microglial activation in the pioglitazone-treated groups versus the placebo group (*ANOVA; *P *= 0.018).

Reactive neuroinflammation was evaluated using CD68 (marker for microglia/macrophages) and GFAP (astrocyte marker) immunostainings. Qualitative observations of CD68 (Figure 7) immunostained brain coronal sections of placebo-treated subjects revealed the presence of numerous CD68 positive cells in the ventrolateral caudate, ventromedial putamen, and the ventral tier of the SN ipsilateral to MPTP administration. Most of these cells exhibited characteristic hyperramified morphology of activated microglia, and many clustered together. In contrast to placebo-treated animals, pioglitazone-treated monkeys displayed a dose-dependent CD68 immunoreactivity, characterized by mildly active microglia ipsilateral to MPTP administration. Quantification showed a significant increase in CD68-ir particle number in the SN ipsilateral to MPTP administration compared to the contralateral side (Wilcoxon Signed Rank test; z = -3.516, *P *< 0.01). A significant difference between treatment groups was found in the side ipsilateral to the MPTP lesion (ANOVA; F [2,13] = 3.778, *P *< 0.05). Multiple comparisons revealed that monkeys treated with 5 mg/kg of pioglitazone had significantly less CD68-ir particle counts in the ipsilateral SN (746 ± 142) compared to placebo (1,331 ± 152) animals (Figure 7; ANOVA Fisher's LSD *P *= 0.018). In addition, there was a significant difference in the total area covered by the particles between ipsi and contralateral sides (Wilcoxon Signed Ranks test; z = -3.516, *P *< 0.01, two-tailed) and a significant difference between treatment groups (ANOVA F [2.13] = 4.248, *P *= 0.038). Multiple comparisons analysis revealed that animals treated with 5 mg/kg of pioglitazone had a significantly smaller CD68 immunoreactive area compared to controls (ANOVA; Fisher's LSD *P = *0.015).

GFAP-ir was significantly increased in the SN on the side ipsilateral to MPTP administration compared to the contralateral side (Wilcoxon Signed Ranks test; z = -2.840, *P *= 0.005 two-tailed), however no significant differences were found between treatment groups (ANOVA; F [2,11] = 0.495, *P = *0.622).

Quantification of nitrotyrosine-ir (NO-dependent oxidative stress marker) showed significantly higher levels in the ipsilateral SN compared to the contralateral side (Wilcoxon Signed Rank test; *P *< 0.001). A trend was revealed toward less intensity in the pioglitazone-treated monkeys compared to placebo (ANOVA; F [2,13] = 3.079, Fisher's LSD *P = *0.068).

Quantification of HO-1-ir (oxidative stress marker) did not show significant differences between ipsilateral vs. contralateral SN (Wilcoxon Signed Rank test; *P *= 0.569) or between treatment groups (ANOVA; F [2,13] = 0.555, *P = *0.407).

The number of TH-ir nigral cells inversely correlated with CD68-ir (Pearson's correlation; *r^2 ^*= 0.279, *P = *0.014), and nitrotyrosine-ir (*r^2 ^*= 0.387, *P *= 0.001) suggesting that the number of surviving DA cells are affected by inflammation and oxidative stress byproducts (Figure 6). Interestingly, CR and FMS test also correlated with CD68 staining intensity (*r^2 ^*= 0.663, *P = *0.007 and *r^2 ^*= 0.697, *P = *0.003, respectively).

### Oral administration of pioglitazone crosses the blood brain barrier

Oral dosing to normal animals of 5 mg/kg of pioglitazone induced higher levels in plasma (mean ± SEM; 1,741.97 ± 317.16 ng/ml) than 2.5 mg/kg (1,360.53 ± 208.01 ng/ml). Yet, in four of the five monkeys, 7.5 mg/kg (1,587.24 ± 413.78 ng/ml) dosing did not result in consistently higher increases of pioglitazone plasma levels compared to 5 mg/kg (repeated measures ANOVA; F [2,6] = 1.344, *P = *0.329).

CSF levels of pioglitazone compared to plasma showed more individual variability. A dose of 2.5 mg/kg (25.08 ± 3.52 ng/ml) induced non-detectable levels in two of the five animals. Both, 5 and 7.5 mg/kg induced consistent measurable levels in CSF. Four of the five monkeys had higher CSF levels of pioglitazone after receiving 5 mg/kg (50.53 ± 23.92 ng/ml) compared to 7.5 mg/kg (17.09 ± 6.22 ng/ml) (repeated measures ANOVA; F [2,4] = 0.206, *P = *0.822).

## Discussion

The present study indicates that oral dosing of pioglitazone to rhesus monkeys after a single intracarotid artery injection of MPTP ameliorates the functional and anatomical consequences of the neurotoxin. Pioglitazone dosing was well tolerated and consistently higher levels were found in plasma and CSF after 5 mg/kg p.o.

To maximize the value of this report for clinical translation, the dosing selected for the efficacy study (2.5 or 5 mg/kg) was equivalent to the ones in use to treat diabetic conditions. This range varies according to the species. Mice (20 mg/kg), or rats (3-10 mg/kg) require higher doses for pioglitazone to be effective [29,30] compared to adult rhesus monkeys (1-3 mg/kg and up to 9 mg/kg) and diabetic patients (15, 30, or 45 mg tablets) [1,31].

Determining when to initiate neuroprotective therapies is critical in defining its success and the magnitude of its effects. Currently we are unable to predict who will develop sporadic PD [22]. The best option in protecting as many DA nigral neurons as possible is to intervene early after the onset/diagnosis of the disease. In this study we started pioglitazone administration soon after MPTP challenge, attempting to resemble clinical conditions of ongoing degeneration in early PD [21,22]. In rhesus monkeys, a single intracarotid artery MPTP infusion immediately induces extensive unilateral depletion of striatal DA (and associated hemiparkinsonian signs, that allow for the selection and matching of the animals according to disability) but little loss of TH-ir nigral neurons[32,33]. By 2-3 months, there is extensive loss of both striatal terminals and nigral neurons. The established syndrome has been shown to persist (without spontaneous recovery) for up to 8 years [20]. This pattern of neurodegeneration creates a window of opportunity to test the efficacy of neuroprotective strategies (e.g.: [20,21]). The placebo-treated monkeys' behavioral stability overtime of the PD syndrome and associated severe loss of DA markers and Nissl-stained nigral cell counts confirm that the criteria used for the selection of the animals was appropriate. Moreover, the Nissl cell counts confirm that the differences between treatment groups in DA markers were related to neuroprotection, not to up- or down- regulation of nigral neuronal phenotype.

A caveat for this experimental design is that in monkeys, pioglitazone achieves a steady-state at 5-7 days [1] which limited the availability of the compound between early dosings. This may have restricted the behavioral and nigrostriatal recovery, inducing improvements mostly in CR (which is more sensitive to small DA changes than FMS) associated to the partial DA nigrostriatal recovery. Yet, we chose this paradigm and its limitations (rather than intervening before MPTP) due to its clinical relevance.

Similar to PD patients, MPTP-intoxicated monkeys present reactive inflammation in the nigrostriatal system that persists for many years [15,16]. In our study, pioglitazone-treated monkeys had a dose-dependent, significantly less CD68 expression than placebo animals, suggesting that pioglitazone decreased the MPTP-induced inflammatory response. This is the first report of pioglitazone (or any PPARγ agonist) inducing an anti-inflammatory response in parkinsonian monkeys, and it agrees with similar findings in other paradigms. For example, pioglitazone prevented microglia-mediated lipopolysaccharide (LPS)-induced cell death in cortical neuron-glia co-cultures as well as in rats with intrastriatal injections of LPS [10,34]. In MPTP-treated mice, pioglitazone decreased glial activation and nitrotyrosine accumulation and induced DA nigral protection [12,13]. Although it is not clear if pioglitazone's effect on inflammatory cells is PPARγ-dependent or independent, our data supports the concept that pioglitazone can modulate glia activation and its harmful effects [35].

We found some evidence of less nigral oxidative stress in pioglitazone-treated monkeys compared to controls. This was observed as a trend toward a decreased expression of nitrotyrosine positive cells and a negative correlation between nitrotyrosine immunostaining and the number of TH-positive nigral neurons. Pioglitazone may have diminished oxidative stress and supported cell survival indirectly by decreasing microglia activation and its harmful metabolites and/or attenuating oxidative stress. It should be noted, however, that since our animals were euthanized 3 months after MPTP administration, this may have affected the expression levels of these markers, compared to microglia immunoreactivity that can persist for many years. Further studies are currently ongoing to assess the effects of PPARγ agonists in oxidative stress.

Pioglitazone neuroprotective effects may have also been related or enhanced by mechanisms different from anti-inflammatory or anti-oxidative pathways. These mechanisms could have triggered early cell recovery and prevented or decreased the inflammatory response. For example, pioglitazone may have facilitated neuropreservation by its effects on glucose metabolism. This compound is currently used clinically as an anti-diabetic agent by increasing insulin sensitization and modulating glucose receptors in peripheral tissue [36,37]. MPTP-induced neurodegeneration increases glucose requirements in the substantia nigra pars compacta of rodents and primates [38,39]. Although we did not observe changes in glucose blood levels in the animals that received pioglitazone, it is possible that there was a functional increase in CNS insulin sensitization, which may have increased the availability of intracellular glucose in a moment of high request for energy by nigral neurons.

MPTP toxicity depends on its transformation by intracerebral MAO-B into its metabolite MPP^+^. Pioglitazone may induce neuroprotection by inhibiting MAO-B activity (and in consequence, decrease toxic MPP^+ ^levels [40]). This possibility is very small in our study, as the majority of MPTP metabolism is estimated to occur during the first few hours after exposure [41-43] and the monkeys started receiving pioglitazone 24 hrs post-MPTP administration. Although MAO-B inhibition may not have affected our results, it should be noted that this property resembles the activity of selegiline and rasagiline, which are widely used in the clinic as anti-parkinsonian treatments [44,45].

Pioglitazone's properties prompted preclinical studies not only in rodent models of PD [12,13], but also Alzheimer disease [46], multiple sclerosis [47,48], amyotrophic lateral sclerosis [49], epilepsy [50], stroke [51,52], and spinal cord injury [53]. In the different models pioglitazone showed capacity to induce neuroprotective and/or restorative effects, mainly associated to modulation of inflammation.

Clinically, pioglitazone has been tested for Alzheimer disease [54-56], multiple sclerosis [57,58], autism [59], stroke [60], amyotrophic lateral sclerosis [61], and Friedreich's Ataxia [62]. The results of these small clinical trials suggest that its administration can benefit patients with neurological disorders. In the case of PD, pioglitazone's clinical evaluation is supported by the reports on its capacity to modulate inflammation, oxidative stress, and glucose uptake, as well as its ability to inhibit MAO-B. Our current results further support the testing of pioglitazone as a disease modifying strategy for early PD.

## Conclusions

This is the first report in nonhuman primates that daily oral administration of the PPAR-γ agonist, pioglitazone, is neuroprotective in a paradigm resembling early PD. Findings include modest but significant improvements in clinical rating, fine motor skills, and preservation of DA striatal fibers and nigral neurons. These changes were associated with a significant attenuation of CD68 positive microglia/macrophages. Significant correlations between behavioral and morphological outcomes were found. A separate experiment confirmed the ability of pioglitazone to cross the blood brain barrier. These results validate PPAR-γ as a target to prevent neurodegeneration in early PD.

## List of Abbreviations

(CR): clinical rating; (DA): dopamine; (FMS): fine motor skill; (GFAP): glial fibrillary acidic protein; (HO-1): heme-oxygenase-1; (MPTP): 1-methyl-4-phenyl-1,2,3,6-tetrahydropyridine; nitrotyrosine (OD): optical density; (PD): Parkinson's disease; (PPAR-γ): peroxisome proliferator activated receptor-gamma; (SN): substantia nigra; (SPE): solid phase extraction; (T2DM): type 2 diabetes mellitus; (TH): tyrosine hydroxylase; (VMAT-2): vesicular monoamine transporter-2.

## Competing interests

The authors declare that they have no competing interests.

## Authors' contributions

CRS performed stereological cell counts, OD and particle data acquisition and analysis, general data analysis and manuscript writing, VJ performed *in vivo *data acquisition and analysis, VB developed and performed immunohistochemistry procedures, KB performed MPTP surgeries and was in charge of overall evaluation of animal health, HAS performed necropsies and analyzed autopsy data, TEZ analyzed pioglitazone levels in plasma and CSF, JWK, assessed the metabolic effects of pioglitazone, JAJ assisted in the experimental design, concept development and data interpretation, and MEE was in charge of the experimental design, concept development, data acquisition and analysis, and manuscript writing. All authors have read and approved the final version of this manuscript.
